# Baseline IgG-Fc N-glycosylation profile is associated with long-term outcome in a cohort of early inflammatory arthritis patients

**DOI:** 10.1186/s13075-022-02897-5

**Published:** 2022-08-25

**Authors:** Thomas Sénard, Irini Flouri, Frano Vučković, Garyfalia Papadaki, Panagiota Goutakoli, Aggelos Banos, Maja Pučić-Baković, Marija Pezer, George Bertsias, Gordan Lauc, Prodromos Sidiropoulos

**Affiliations:** 1 Center for Proteomics and Metabolomics, Leiden University Medical Center, 2300 RC Leiden, The Netherlands; 2 Rheumatology and Clinical Immunology, University Hospital of Heraklion, 71003 Heraklion, Greece; 3 Genos Glycoscience Research Laboratory, Zagreb, Croatia; 4 Laboratory of Rheumatology, Autoimmunity and Inflammation, Medical School, University of Crete, 71305 Heraklion, Greece; 5 Laboratory of Inflammation and Autoimmunity, Biomedical Research Foundation of the Academy of Athens, 11527 Athens, Greece; 6 Faculty of Pharmacy and Biochemistry, University of Zagreb, Zagreb, Croatia

**Keywords:** Rheumatoid arthritis, Immunoglobulin G, Fragment crystallizable, *N*-glycosylation, Inflammation

## Abstract

**Background:**

Rheumatoid arthritis (RA) is a chronic autoimmune disease for which prediction of long-term prognosis from disease’s outset is not clinically feasible. The importance of immunoglobulin G (IgG) and its Fc *N*-glycosylation in inflammation is well-known and studies described its relevance for several autoimmune diseases, including RA. Herein we assessed the association between IgG *N*-glycoforms and disease prognosis at 2 years in an early inflammatory arthritis cohort.

**Methods:**

Sera from 118 patients with early inflammatory arthritis naïve to treatment sampled at baseline were used to obtain IgG Fc glycopeptides, which were then analyzed in a subclass-specific manner by liquid chromatography coupled to mass spectrometry (LC-MS). Patients were prospectively followed and a favorable prognosis at 2 years was assessed by a combined index as remission or low disease activity (DAS28 < 3.2) and normal functionality (HAQ ≤ 0.25) while on treatment with conventional synthetic DMARDs and never used biologic DMARDs.

**Results:**

We observed a significant association between high levels of IgG2/3 Fc galactosylation (effect 0.627 and adjusted *p* value 0.036 for the fully galactosylated glycoform H5N4F1; effect −0.551 and adjusted *p* value 0.04963 for the agalactosylated H3N4F1) and favorable outcome after 2 years of treatment. The inclusion of IgG glycoprofiling in a multivariate analysis to predict the outcome (with HAQ, DAS28, RF, and ACPA included in the model) did not improve the prognostic performance of the model.

**Conclusion:**

Pending confirmation of these findings in larger cohorts, IgG glycosylation levels could be used as a prognostic marker in early arthritis, to overcome the limitations of the current prognostic tools.

**Supplementary Information:**

The online version contains supplementary material available at 10.1186/s13075-022-02897-5.

## Background

Rheumatoid arthritis (RA) is a common systemic autoimmune disease that causes primarily chronic joint inflammation and functional limitation [1]. Major steps in understanding disease’s pathogenesis have been accomplished, revealing several genetic loci, epigenetic mechanisms and environmental factors involved in breaching immune tolerance for disease initiation and progression [2]. Auto-immunity to post-translationally modified proteins [citrullinated (ACPAs), carbamylated (anti-CarP Abs)] as well as the development of rheumatoid factors (RFs) which recognize immunoglobulin G (IgG) Fc fragments, start in the preclinical stage of the disease [3, 4]. Several mechanisms mostly unknown, contribute to the progression to clinically apparent synovitis, a stage when the patient firstly present to the clinic [5]. Physicians evaluating for the first time patients with inflammatory arthritis have to make a diagnosis and assess the prognosis. Diagnosis of RA is still mainly based on clinical data, while laboratory tests like RA-specific autoantibodies and acute-phase reactants, assist in the diagnosis [6]. Although autoantibodies (RFs and ACPAs) can be present years before the disease appears [7], they have limitations as diagnostic tools since healthy persons may also develop mostly RF, and recent epidemiological studies have shown an increased incidence (up to 50%) of RF and ACPA seronegative RA [8, 9]. Interestingly, patients not fulfilling the classification criteria for RA but having inflammatory arthritis and no evidence of another systemic autoimmune disease are classified as undifferentiated arthritis (UA) and treated accordingly [10]. The second challenge is to assess the prognosis of early RA patients. Most studies based on clinical, serological, and baseline radiological data, revealed that the presence of RF or ACPAs, presence of bone erosions, and increased acute phase reactants predict a more aggressive disease [11]. Nevertheless, the accuracy of the currently proposed models for predicting joint damage or response to therapy has limitations [12, 13]. Thus, there is a need for novel diagnostic and prognostic biomarkers. Along this concept, there are recent data supporting the value of high-throughput molecular data at the single-cell level as prognostic tools for disease’s outcome [14, 15].

RF and ACPA of IgG isotype belong to the family of glycoproteins. Their fragment crystallizable (Fc)-region, as well as seldomly the antigen-binding (Fab)-regions, are known to be *N*-glycosylated. The *N*-glycosylation profile is essential for antibodies’ effector and antigen binding functions and was shown to depend on different factors such as genetics, epigenetics, aging, and pathological states [16–18]. Studies of total serum IgG *N*-glycosylation from RA patients demonstrated a lower galactosylation, which was associated to disease activity, but also predicted the patients’ response to therapy and preceded disease onset by up to several years [19–24]. Moreover, glycosylation of antigen-specific IgGs, such as ACPA, was shown to differ from total IgGs in both Fc and Fab regions, thus influencing its functional activities [25–27]. This stresses the importance of IgG glycosylation to RA pathogenesis. Interestingly, mouse studies have shown that IL-23-activated TH17 cells accumulating in germinal centers during the prodromal phase of experimental arthritis, can alter the glycosylation profile of IgGs toward a pro-inflammatory autoantibody repertoire and trigger the onset of experimental arthritis [28]. A previous small study from Lundström et al. revealed changes in IgG-Fc galactosylation in the early stage of RA as well as its possible predictive power on the treatment response [29].

In the current study, we evaluated the value of total serum IgG Fc *N*-glycosylation as a diagnostic and prognostic biomarker of patients with early arthritis. We applied a state-of-the-art liquid chromatography–mass spectrometry (LC-MS) based workflow for the analysis of subclass-specific IgG Fc *N*-glycosylation in the sera of 118 early, naïve to treatment, inflammatory arthritis patients. Patients were cross-sectionally sampled at baseline and prospectively followed for 2 years. A diagnosis of RA or UA was based on established classification criteria, while the long-term prognosis of the disease was assessed during 2 years of follow-up.

## Methods

### Materials

Formic acid (FA) was purchased from Merck (Darmstadt, Germany). Ammonium bicarbonate (ABC) was acquired from Acros Organics (Pittsburgh, PA, USA). Trifluoroacetic acid (TFA) was obtained from Sigma-Aldrich (St Louis, MI, USA). LC-MS grade acetonitrile (ACN) was purchased from Honeywell (Morris Plains, NJ, USA). Sequencing grade trypsin was obtained from Promega (Fitchburg, WI, USA).

### Patients cohort—outcomes

The “Early Arthritis Clinic” of the University Hospital of Heraklion is a prospective cohort of patients with inflammatory arthritis and its details were previously described by Fanouriakis et al. [13]. Briefly, patients were assessed at baseline and in regular intervals up to 2 years. For the present study, we selected a group of patients naïve to any immunosuppressive treatments with available serum at baseline evaluation (*n*=118). At baseline, demographics, RA clinical characteristics [disease activity assessed by 28 joint counts (DAS28), functionality assessed by “Health Assessment Questionnaire (HAQ-DI)], and laboratory tests [autoantibodies (RF and/or ACPA)] were also recorded (Table 1). The patients were prospectively followed for 2 years, with clinical, laboratory, and disease-related treatments documented. Treatment decisions were made by treating rheumatologists, according to national guidelines, while no strict treat-to-target protocol was followed. The study was approved by the Institutional Review Board of the University Hospital of Heraklion, Crete (decision number: 1476/20-03-2012), and all patients provided written informed consent for their participation.

**Table 1:** Baseline demographics, clinical characteristics, and patient treatments at the end of follow-up (24 months)

Characteristics	RA and UA patients (***n*** = 118)
Age (mean ± SD)	53 ± 15.66
Gender (% females)	80.5
Symptoms duration (weeks mean ± SEM)	53.79±8.72
RF positive (%), mean (SD)	8.4, [35.6 (167.9)]
ACPA positive (%), mean (SD)	16, [21.9 (50.8)]
ESR (mean ± SEM, mm/h)	30.46 ± 2.36
CRP (mean ± SEM, mg/dL)	1.96 ± 0.35
Disease activity score 28 joints (DAS28) (mean ± SEM)	4.85 ± 0.14
**Treatments during 24 months of follow-up,*N*(% of all patients)**	
Biologic DMARD +/− csDMARDs	16 (13.6)
Methotrexate	45 (38.1)
Leflunomide	26 (22)
HCQ	31 (26.3)
Corticosteroids per os	11 (9.3)

*RF* rheumatoid factor, *ACPA* anti-citrullinated protein antibodies, *ESR* erythrocyte sedimentation rate, *CRP* C-reactive protein, *DMARD* disease-modifying anti-rheumatic drug, *HCQ* hydroxychloroquine

Concerning the outcomes for the present analysis, a diagnosis of RA or UA was done either at the baseline evaluation or whenever during the follow-up, based on established criteria [6]. Moreover, aiming to assess long-term outcomes (at 2 years) and in order to overcome the limitation of missing data, we formulated 3 composite long-term outcomes of clinical significance (Table 2): (1) initiation of biological disease-modifying antirheumatic drug (bDMARD) at any time during follow-up (adverse outcome 1), (2) presence of high disease activity (HDA) (based on DAS28 > 5.1) or compromised function (based on HAQ > 1.0) at 2 years while on treatment with conventional synthetic DMARDs (csDMARDs) (except hydroxychloroquine (HCQ) or initiation of bDMARDs (adverse outcome 2), (3) remission or low disease activity (based on DAS28 < 3.2) and normal functionality (based on HAQ ≤ 0.25) while on treatment with csDMARDs and never use bDMARDs (favorable outcome).

**Table 2:** Different outcomes assessed for the statistical analysis

Outcomes	Conditions	Data availability
Adverse 1	Treatment with bDMARD within 2 years	118/118
Adverse 2	HAQ > 1 or DAS28 > 5.1 while on csDMARDs or treatment with bDMARD at 2 years	94/118
Favorable	DAS28 < 3.2 and HAQ ≤ 0.25 and no treatment with bDMARD at 2 years	93/118

Samples and in-house serum standards were randomized into two 96-well plates prior to IgG isolation.

### IgG isolation

IgGs were captured from serum by affinity chromatography using 96-well protein G monolithic plate (BIA Separations, Ajdovščina, Slovenia) as previously described by Pučić et al. [30]. Briefly, 100 μL of serum was diluted with 700 μL of 1X phosphate-buffered saline (PBS) and filtered through a 0.45μm GHP filter plate (Pall Corporation, Ann Arbor, MI, USA). Samples were then loaded onto a protein G plate and washed three times with 2 mL of 1X PBS. IgGs were eluted with 1 mL of 0.1 M FA and neutralized with 170 μL of 1 M ABC. The protein concentration was measured using a NanoDrop 8000 spectrophotometer (Thermo Fisher Scientific, Waltham, MA, USA).

### IgG digestion and purification

After isolation, an aliquot of isolated IgGs (ranging from 7.44 to 46 μg) was digested with 0.18 μg of sequencing grade trypsin overnight at 37 °C. The tryptic digests were cleaned-up by reverse-phase solid-phase extraction (RP-SPE) using Chromabond C18 beads (Macherey-Nagel, Düren, Germany). In short, samples were diluted ten times with 0.1 % TFA, loaded onto beads, and then washed three times with 0.1 % TFA. Glycopeptides were eluted from the beads using 20 % ACN and dried down by vacuum centrifugation.

### RPLC-MS analysis of IgG-Fc glycopeptides

The obtained glycopeptides were reconstituted in 20 μL ultrapure water before being analyzed by reverse-phase liquid chromatography (RPLC) coupled with mass spectrometry (MS). LC separations were performed on a nanoAcquity UPLC system (Waters, Milford, MA, USA) coupled to a quadrupole time-of-flight (q-TOF) MS instrument (Compact, Bruker, Bremen, Germany) using a CaptiveSpray electrospray ionization (ESI) source from Bruker. Separations were carried out using a C18 analytical column (150 mm x 100 μm ID, 100 Å, Advanced Materials Technology, Wilmington, DE, USA) and the column compartment was set to 30 °C. The samples (10 μL) were loaded first on a C8 trap column (PepMap 100, 5 mm × 300 μm ID, Thermo Fisher Scientific) at a flow rate of 15 μL/min. The separations were achieved using mobile phases which consisted in 0.1 % TFA for solvent A and 80 % ACN, 0.1 % TFA for solvent B. The gradient was from 18 % to 28 % of solvent B in solvent A at a flow rate of 1 μL/min for 8 min.

The mass spectrometer was operated in positive-ion mode with electrospray voltage set to 1.5 kV. The ESI source was used with a CaptiveSpray nanobooster to add a dopant enriched nitrogen in combination with ACN at a pressure of 0.2 bar as well as a drying gas of nitrogen set to 4 L/min at 150 °C. The quadrupole collision cell energy was 5 eV. Mass spectra were recorded in a *m/z* range of 800–2000.

LC-MS data were treated with a specific software (LacyTools version 1.1.0) as previously described [31] with minor changes such as the minimum features for alignment was set to 5 and the number of data points was 33. To compare measurements across samples and calculate the relative abundances of each IgG isoform, the following normalization method was performed. First, the isotopic pattern was corrected for each glycopeptide by summing the intensities at charge states 2+ and 3+ and dividing them by the fraction of the isotopic pattern found by the software. Then, the relative abundance of each glycopeptide was calculated by dividing the corrected sum by the total intensity of each peak. The relative abundance is expressed in % of total normalized intensity and represents the relative abundance of the given glycopeptide within the sum of all glycopeptides recorded for a single subclass. This treatment resulted in relative abundance data of 16 IgG1, 9 IgG2/3, which share the same tryptic peptide sequence, and 10 IgG4 Fc *N*-glycoforms available for each sample.

The robustness of the method was assessed by calculating the average relative abundances, the standard deviations (SD) and the coefficients of variation (CV) from all the glycoforms of each IgG subclass of 7 serum standards. The median CVs were 3.74% for IgG1, 4.88% for IgG2/3, and 4.10% for IgG4 (Supplementary Table 1).

### Statistical analysis

The results of subclass-specific IgG Fc *N*-glycoprofiling were then subjected to statistical analysis via a general linear model (ANCOVA) and corrected for age, sex, and duration of symptoms. Prior to analyses, glycan variables were all transformed to a standard normal distribution (mean = 0, SD = 1) by an inverse transformation of ranks to normality (R package “GenABEL,” function rntransform). Using rank-transformed variables in analyses makes estimated effects of different glycans comparable, as transformed glycan variables have the same standardized variance. False rate discovery was controlled using the Benjamini-Hochberg procedure. As IgGs showed different numbers of glycoforms per subclass and due to the smaller sample size which would make the multiple testing burden too large, only the 4 most abundant species were used in the statistical analysis. These species H3N4F1, H4N4F1, H5N4F1, and H5N4F1S1 can be described by their number of hexose (H), *N* - acetylglucosamine (N), fucose (F) and sialic acid residues (S). Patients were classified into 2 groups, based on their diagnosis, either rheumatoid arthritis (RA, group 1, 60% of patients) or undifferentiated arthritis (UA, group 2, 40% of patients). Three different long-term outcomes were tested, 2 adverse and 1 favorable based on the clinical traits after 2 years (Table 2).

## Results

### Lack of association of IgG glycosylation profiles to diagnosis

LC-MS-based workflow was used to study subclass-specific IgG Fc *N*-glycosylation in 118 early inflammatory arthritis patients sampled before any treatment. LC conditions resulted in the separation of the glycopeptides from IgG1 (EEQYNSTYR), IgG2/3 (EEQFNSTFR), and IgG4 (EEQFNSTYR) (shown in Supplementary Fig. 1). Relative abundances of all glycoforms were obtained from the LC-MS data (Supplementary Fig. 2) before being treated with the LaCyTools software. The statistical analysis was performed using the relative abundances of the four most abundant glycoforms of IgG1, 2/3, and 4.

**Fig. 1:**
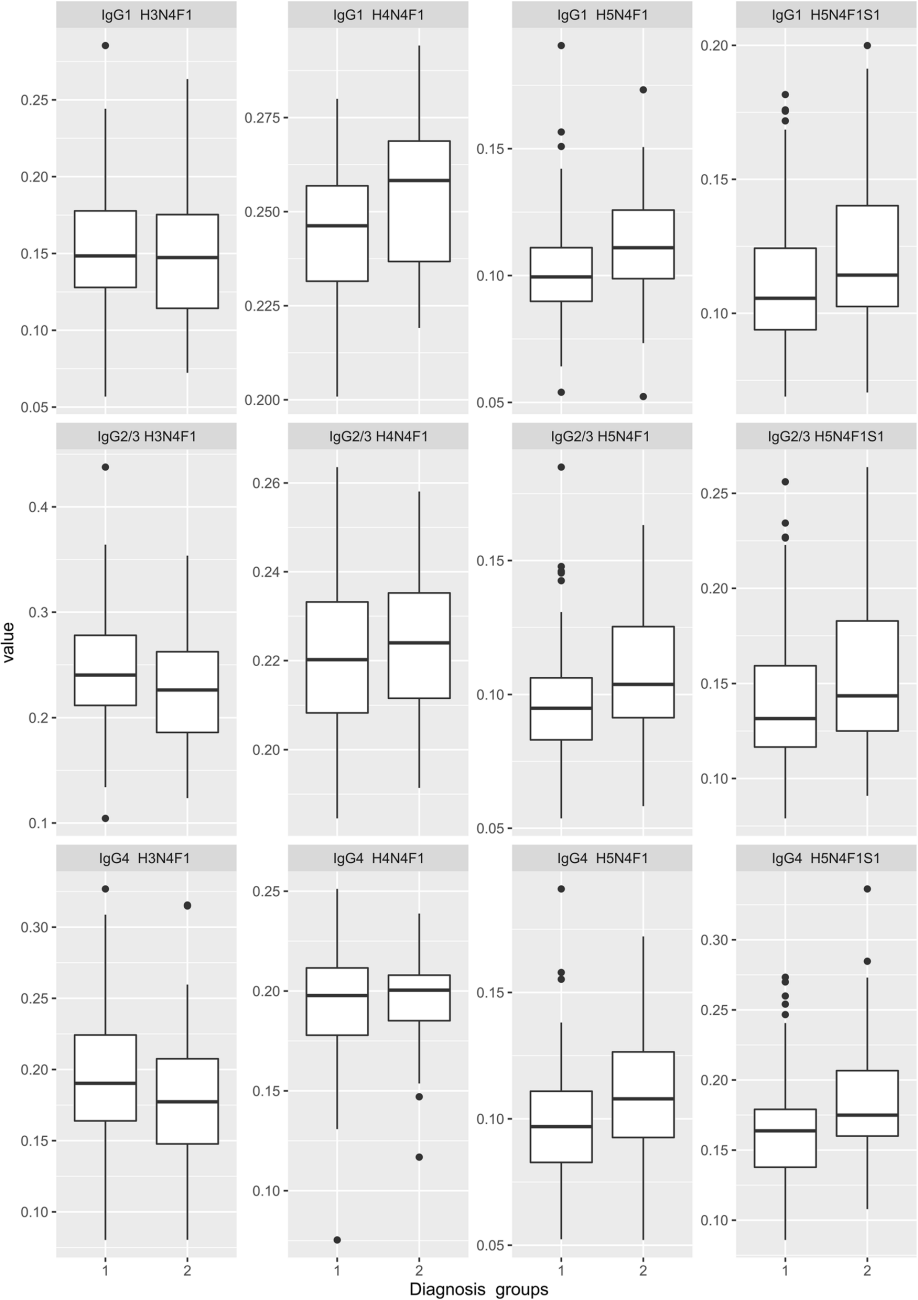
Relative abundances of subclass-specific IgG Fc *N*-glycoforms (H3N4F1, H4N4F1, H5N4F1, H5N4F1S1) in two diagnosis groups: 1 – rheumatoid arthritis; 2 – undifferentiated arthritis. No statistically significant differences between the groups using a general linear model as specified in the Methods section. Glycan compositions are given in terms of Hexose (H), *N*-acetylglucosamine (N), fucose (F), and sialic acid residues (S). The data were presented as a box plot where the lower and upper hinges correspond to the first and third quartiles. The upper whisker extends from the hinge to the largest value no further than 1.5 times the inter-quartile range (IQR) from the hinge while the lower one extends to the smallest value at most 1.5 times IQR. Data beyond the end of the whiskers are outliers and are plotted individually. The value represents the relative abundance of each presented glycoform within the sum of all glycoforms on a single subclass

At first, we looked at the differences in the relative abundances of the 4 most abundant glycoforms (H3N4F1, H4N4F1, H5N4F1, and H5N4F1S1) between the 2 diagnostic groups, namely RA and UA (Fig. 1). Even though patients diagnosed with early undifferentiated arthritis (group 2) had higher relative abundances of galactosylated and sialylated *N*-glycoforms (H4N4F1, H5N4F1, and H5N4F1S1) in all IgG subclasses compared to rheumatoid arthritis patients (group 1), none of the differences reached significance threshold (Supplementary Table 2).

### IgG glycosylation profile predicts long-term outcome

**Fig. 2:**
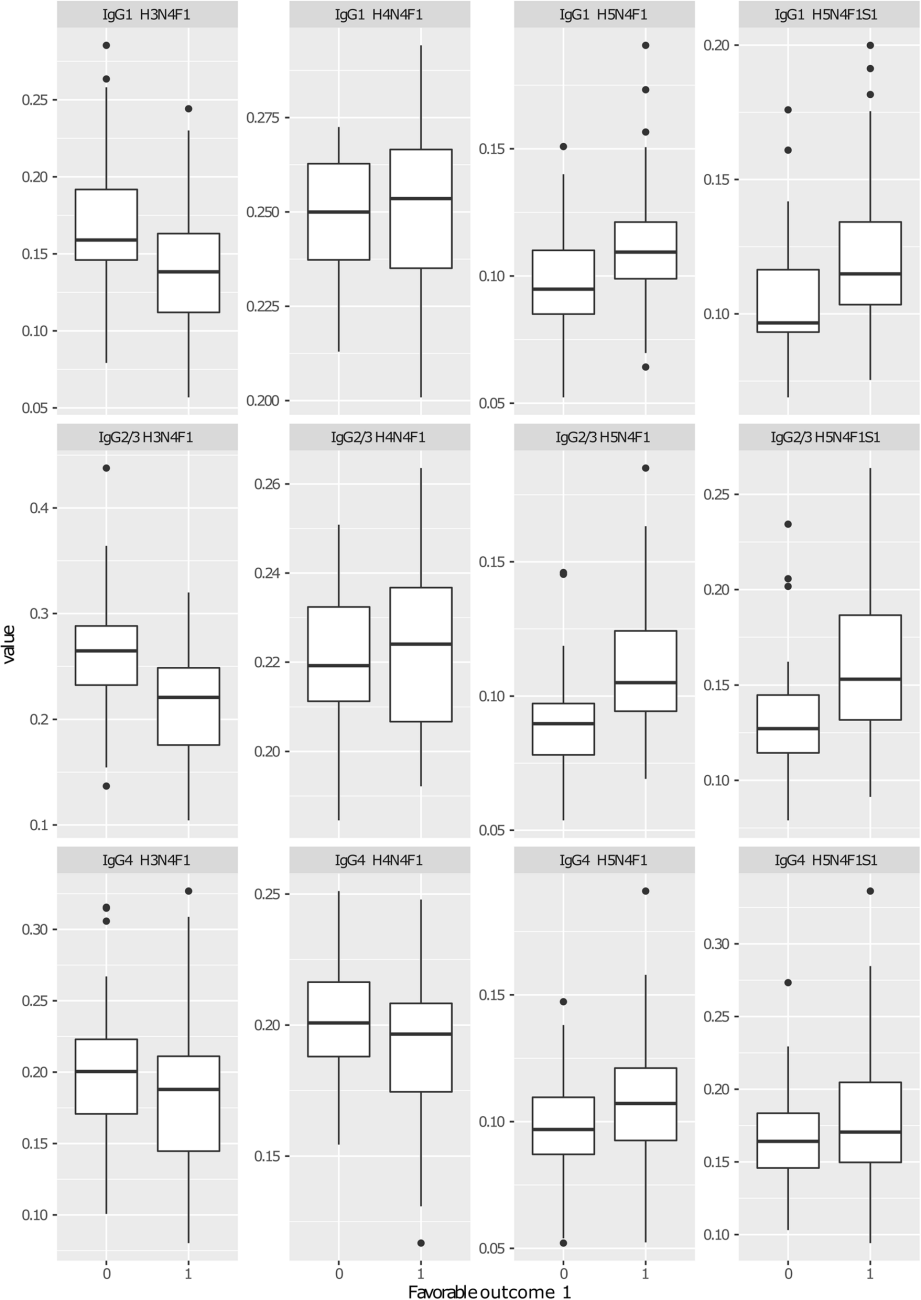
Relative abundances of subclass-specific IgG Fc *N*-glycoforms (H3N4F1, H4N4F1, H5N4F1, H5N4F1S1) in patients without (0) or with (1) “Favorable outcome” after 2 years of treatment. “Favorable outcome” was defined as DAS28 < 3.2, HAQ ≤ 0.25, and no biological disease-modifying antirheumatic drug was received during treatment. *Statistically significant associations (adjusted *p* value < 0.05) following the general linear model described in the Methods section. Glycan compositions and box plot description as in Fig. 1

Since the two diagnosis groups showed no statistically significant differences in glycosylation profiles, they were grouped together for further analysis of the association between IgG *N*-glycoforms and disease outcomes defined in three different groups (adverse outcomes 1 and 2, and favorable outcome; Table 2). The analysis revealed a significant association between IgG2/3 Fc *N*-glycoforms describing galactosylation levels (H3N4F1 and H5N4F1) and “favorable outcome” (defined as DAS28 < 3.2 and HAQ ≤ 0.25 and no treatment with bDMARD at 2 years) (Supplementary Table 3). Patients with a higher pre-treatment galactosylation (higher abundance of H5N4F1) and hence a lower pre-treatment agalactosylation of IgG2/3 (lower abundance of H3N4F1) showed a favorable outcome after 2 years of treatment (Fig. 2). Sialylation of the same subclass (IgG2/3 H5N1F1S1) as well as galactosylation and sialylation of both IgG1 and IgG4 seemed to follow the same trend but the adjusted p-values were not significant (Supplementary Table 3). Interestingly, when we analyzed baseline glycosylation levels with individual parameters (i.e., DAS28 at 24 months, HAQ at 24 months, use of biologics ever) we found no statistical association, apart from a trend of association between IgG1 (H5N4F1S1) and HAQ difference between baseline and 24 months (*p* adj=0.087).

Next, we checked for associations between clinical traits at baseline, such as seropositivity (i.e., the presence of RF and/or anti-CCP antibodies), DAS28 (low < 3.2 versus medium-high ≥ 3.2) HAQ (mild-moderate compromised function < 1 versus severe disability ≥ 1), and the outcomes after 2 years. While HAQ was associated with “adverse outcomes 1” and “2”, DAS28 was associated with “favorable outcome” (Supplementary Table 4). The correlation between the glycoforms of IgG subclasses and the baseline clinical traits, acute phase reactants, and autoantibody levels (RF and ACPA levels) was also tested but did not show any statistically significant associations (Supplementary Table 5). Finally, in a multivariate analysis to predict outcome (with HAQ, DAS28, RF, and ACPA included in the model), the inclusion of IgG glycoprofiling did not improve the prognostic performance of the model (data not shown).

## Discussion

In the present study, we applied a validated and high-throughput subclass-specific IgG Fc *N* - glycosylation LC-MS profiling method, to evaluate IgG glycosylation status as a diagnostic and prognostic tool in early inflammatory arthritis patients [32]. Moreover, with regard to sample processing and data analysis, this method has also been optimized to minimize the batch effect as well as other effects that can occur during the analysis of a large number of samples. This state-of-the-art method has been widely recognized as a powerful diagnostic tool for the analysis of IgG glycosylation [33, 34]. Thus, after quantification of several glycoforms, corrected for baseline factors which may affect IgG glycosylation (symptoms duration, age, and sex) we found glycosylation profiles to be associated with 2 years’ prognosis [30, 35]. Of note, the serum samples used in this study were derived from treatment-naïve patients; therefore, variations in IgG glycosylation do not represent any therapeutic effect but may reflect the impact of inflammation.

Approximately 50–70% of the patients with early arthritis progress to RA, while 30–40% have a diagnosis of UA [36]. Thus, the first clinical challenge when assessing patients with early inflammatory arthritis is to establish the diagnosis. For this reason, we aimed to assess the correlation between IgG *N*-glycosylation and the two diagnostic groups. Preliminary statistical analysis did not reveal significant differences in IgG glycosylation between patients with UA and RA. This was not an unexpected finding since there are data supporting a decreased immunoglobulins’ glycosylation profile in inflammatory arthritides, irrespective of the specific diagnosis (RA or spondylarthritis). Moreover, although data comparing levels of glycosylation between different arthritides are not available, it has been shown that effective treatment with TNF inhibitors increased IgG glycosylation levels in both RA and spondylarthritis [37]. Consequently, our data corroborate the above-mentioned data supporting that glycosylation levels are mostly affected by the inflammatory burden and that disease-specific mechanisms are not important contributors.

As previously mentioned, for patients with early inflammatory arthritis—either RA or UA—the second challenge is to predict the long-term prognosis. To address this, we set up 3 major outcomes of clinical importance based on disease activity/function at 24 months and medication used during the 2-year period. We used a combination of clinically significant parameters, since the use of biologics was rather low (16.9% (20/118)) and, most importantly, in clinical practice treatment decisions are not strictly based on disease activity levels for various reasons (both patient- and physician-related) [38–40]. Thus, as it has been shown in several early arthritis cohorts, 35-55% of early arthritis patients are not treated with bDMARDs according to the treat-to-target principle, we considered it valid to have a “combo” outcome including treatment with bDMARDs and high disease activity/compromised function. The analysis showed that, by keeping the two diagnostic groups separated, there were no statistically significant associations between IgG glycoforms and neither adverse nor favorable outcomes. Nevertheless, by combining these two groups together, and thus increasing the statistical power, a significant association was shown between higher levels of digalactosylated IgG glycoforms and favorable outcomes after 2 years of treatment. These results are in line with previous studies as aberrant IgG *N*-glycosylation profile in RA patients has been reported multiple times [22–24]. Lower level of IgG galactosylation was shown to associate with disease activity and symptom severity and even preceded the development of disease [17, 41]. Nonetheless, our study is the first to show an association of IgGs glycosylation profile and long-term outcome. The small number of patients in our study could explain why only the IgG2/3 subclasses express this typical agalactosylation level, as we would expect the same trend for IgG1. Other possible reason could be subtle differences in Fc glycosylation profiles of different IgG subclasses. Thus, we find lower galactosylation and sialylation of IgG2/3 compared to other subclasses, confirming observations from previous studies [42, 43]. These differences in Fc glycosylation reflect in their binding affinity towards Fcγ-receptors (FcγR) and ability to modulate effector functions [44]. According to literature, IgG2 has the lowest affinity for FcγRs and lowest ADCC capacity, while the opposite is true for IgG3 [44–46]. Unfortunately, the used glycoprofiling method did not allow separate identification of IgG2 and IgG3 Fc glycoforms thus hampering the evaluation of their distinct associations with the disease outcome.

Interestingly, in our cohort once again the value of a patient’s functionality (HAQ status) was associated with long-term prognosis. Nevertheless, in the multivariate analysis, glycosylation status was not predictive of the 2 years’ outcome.

A limitation of our study is that we have not had the possibility to assess the biological significance of IgG glycosylation alterations in the context of an autoimmune disease. This is especially true for autoantibodies positive (ACPA or RF) RA. Nevertheless, published literature suggests potential mechanisms of IgG-Fc glycosylation levels contribute to immune responses. Thus, it has been shown that the two Fc-linked carbohydrates interposed between the heavy chains are crucial for the three-dimensional structure and biological activity of the antibody [47]. Data have shown that the removal of the Fc glycans diminishes IgG Fc-mediated biological activity due to the failure of the non-glycosylated molecule to bind to FcγR [48]. Additionally, certain modifications affect IgG-mediated antibody-dependent cellular cytotoxicity (ADCC) [49], while Fc-linked glycans modulate the activation of the complement cascade [50]. Finally, it has been shown that highly galactosylated IgG1 immune complexes (ICs), the inhibitory IgG receptor Fc-gamma RIIB and the C-type lectin-like receptor dectin-1, suppress C5a receptor (C5aR) functions and respective C5a-dependent inflammatory responses [51]. Whether the above mechanisms are important in the context of RA or whether they affect the function of RA-specific autoantibodies is not yet addressed. Notably, and corroborating the possibility of contribution in the inflammatory responses in RA, it has been observed a significant correlation between levels of aberrant IgG galactosylation and disease activity in RA patients [52].

An additional limitation of our study is the lack of sequential sampling of the patients during follow-up. One could argue that this supplementary information could have additive value to the baseline characteristics of IgG *N*-glycosylation status, as a predictor for disease’s outcome. Nevertheless, the analysis would have been more complicated since the potential contribution of other factors while on treatment (infections, aging, treatment effect, etc.), cannot be excluded. Indeed, alterations with total (not antigen-specific) IgG glycosylation have been associated with various physiological and pathological states since they seem responsive to general inflammatory capacity.

## Conclusion

In conclusion, we found IgG2/3 Fc *N*-glycoforms to be associated with a favorable prognosis (low disease activity or remission, preserved functionality, and no treatment with bDMARD) in patients with early arthritis follow-up for 2 years. Should the present data be confirmed in a larger cohort could be of clinical value. Since currently available prognostic tools have significant limitations, further research should aim to develop a predictive tool of high specificity and sensitivity based on the combination of clinical, serological data and novel biomarkers.

## Supplementary Information

**Additional file 1: Supplementary Figure 1**. Example of IgG glycopeptides subclass separation of the H4N4F1 *N*-glycoform. Extracted-ion chromatogram (EIC) of IgG1 (EEQYNSTYR) is represented by the red trace, IgG2/3 (EEQFNSTFR) by the blue trace and IgG4 (EEQFNSTYR) by the green trace. LC-MS conditions: Temperature 30°C. Gradient from 18% to 28% B in 8 min at 1 μL/min. H4N4F1 glycoform is represented using the Symbol Nomenclature for Glycans.

**Additional file 2: Supplementary Figure 2**. Example of MS spectra with their annotated glycoforms for IgG1 (A), IgG2/3 (B) and IgG4 (C). The right side of each panel represents the glycoforms at the charge state +2 while the left side shows the same glycoforms at the charge state +3. The individual glycoforms are represented using the Symbol Nomenclature for Glycans.

**Additional file 3: Supplementary Table 1**. Average relative abundances, standard deviations (SD), coefficients of variation (CV) and median CVs of IgG *N*-glycoforms, from the 7 serum standards, quantified with the LaCyTools software. Relative abundances were calculated following normalization of total intensity as described in the Methods section.

**Additional file 4: Supplementary Table 2**. Association of relative abundances of IgG *N*-glycoforms and the two diagnosis groups. Standard errors, *p*-values and *p*-values adjusted for multiple testing (*p*-value adjusted > 0.05) were calculated following the general linear model with age, sex and duration of symptoms included as additional covariates.

**Additional file 5: Supplementary Table 3**. Association of the relative abundances of IgG *N*-glycoforms of the two diagnosis groups and the different clinical outcomes (“favorable outcome” and “adverse outcomes 1” and “2”). Standard errors, *p*-values and *p*-values adjusted for multiple testing were calculated based on the general linear model with age, sex and duration of symptoms included as additional covariates. Statistically significant associations are described by an adjusted *p*-value < 0.05.

**Additional file 6: Supplementary Table 4**. Association of baseline clinical characteristics and long-term outcomes. Standard errors, *p*-values and p-values adjusted for multiple testing were determined using a general linear model (age, sex and duration of symptoms included as additional covariates) and based on HAQ, DAS28, seropositivity (presence of RF and/or anti-CCP antibodies) at baseline of the two diagnosis groups for the “favorable outcome” and “adverse outcomes 1” and “2”. Statistically significant associations are described by an adjusted *p*-value < 0.05.

**Additional file 7: Supplementary Table 5**. Association of IgG *N*-glycoforms and baseline clinical characteristics (HAQ, DAS28, seropositivity, acute phase reactants and autoantibody levels) of the two diagnostic groups. Standard errors, *p*-values and *p*-values adjusted for multiple testing were calculated following a general linear model (age, sex and duration of symptoms included as additional covariates) and based on the relative abundance of IgG *N*-glycoforms (*p*-value adjusted > 0.05).

## Data Availability

Raw data supporting the research of this article will be made available by the authors, without undue reservation.

## Abbreviations

ABC: Ammonium bicarbonate
ACN: Acetonitrile
ACPA: Anti-citrullinated peptides antibodies
bDMARD: Biological disease-modifying antirheumatic drug
CCP: Cyclic citrullinated peptides
csDMARD: Conventional synthetic disease-modifying antirheumatic drug
CV: Coefficient of variation
DAS28: Disease activity score
ESI: Electrospray ionization
Fab: Fragment antigen-binding
Fc: Fragment crystallizable
FA: Formic acid
HAD: High disease activity
HAQ: Health assessment questionnaire
HCQ: Hydroxychloroquine
IgG: Immunoglobulin G
LC-MS: Liquid chromatography–mass spectrometry
PBS: Phosphate-buffered saline
RA: Rheumatoid arthritis
RF: Rheumatoid factor
SD: Standard deviation
TFA: Trifluoroacetic acid
UA: Undifferentiated arthritis
