# Supplementary figures and images for: Baseline IgG-Fc N-glycosylation profile is associated with long-term outcome in a cohort of early inflammatory arthritis patients
Source: Arthritis Res Ther. 2022 Aug 25;24:206. doi: 10.1186/s13075-022-02897-5 (PMC9404591; doi:10.1186/s13075-022-02897-5)

**
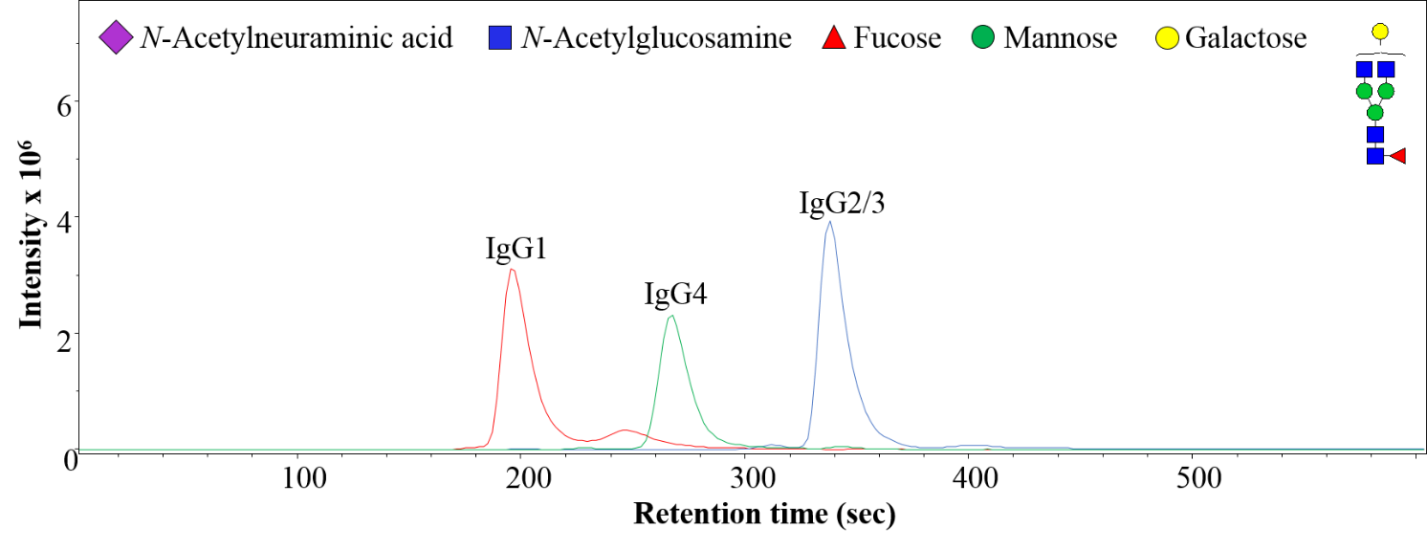
**

Supplement: Supplementary file 1 — Additional file 1: Supplementary Figure 1. Example of IgG glycopeptides subclass separation of the H4N4F1 N-glycoform. Extracted-ion chromatogram (EIC) of IgG1 (EEQYNSTYR) is represented by the red trace, IgG2/3 (EEQFNSTFR) by the blue trace and IgG4 (EEQFNSTYR) by the green trace. LC-MS conditions: Temperature 30°C. Gradient from 18% to 28% B in 8 min at 1 μL/min. H4N4F1 glycoform is represented using the Symbol Nomenclature for Glycans. [file 13075_2022_2897_MOESM1_ESM.docx]

**
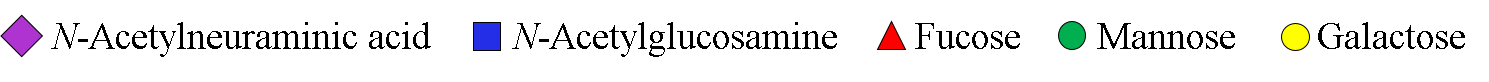

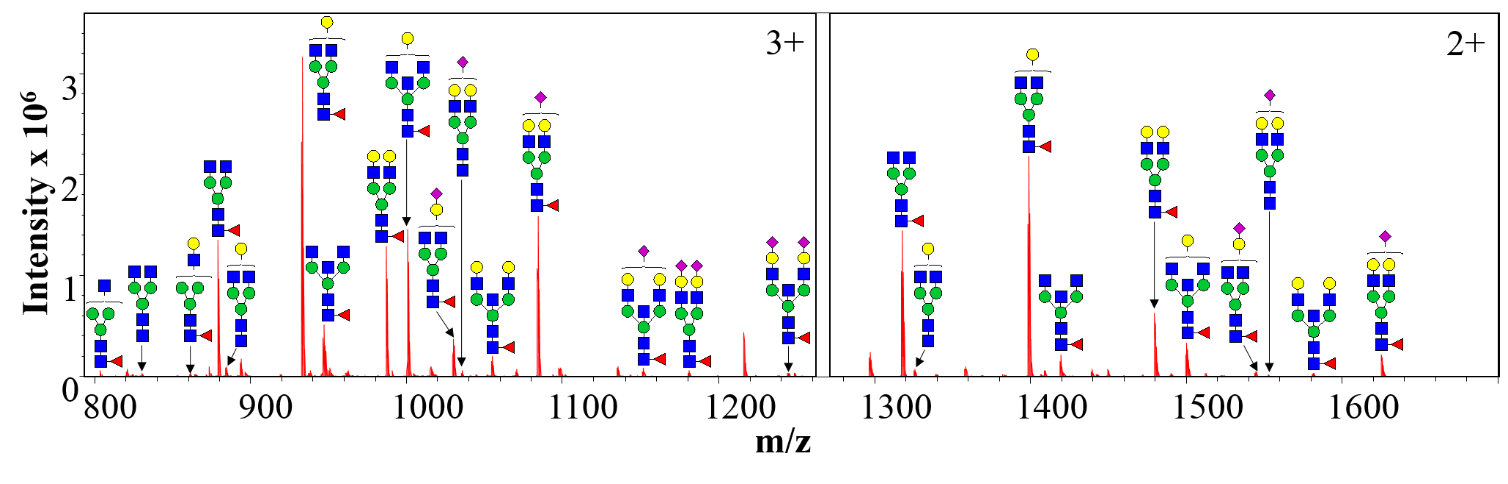

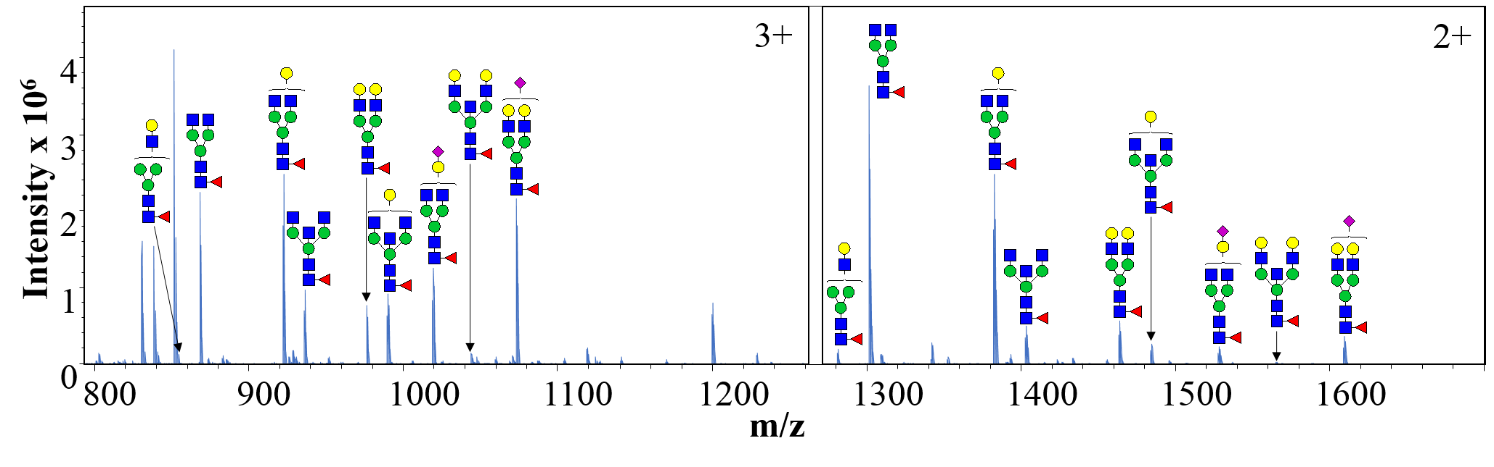

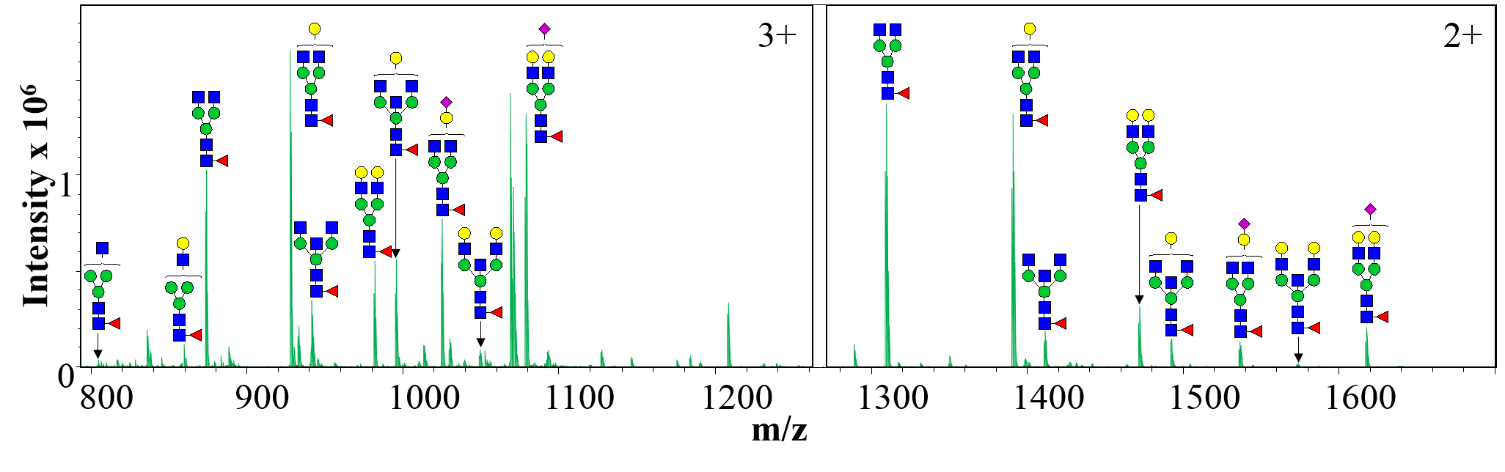
**

Supplement: Supplementary file 2 — Additional file 2: Supplementary Figure 2. Example of MS spectra with their annotated glycoforms for IgG1 (A), IgG2/3 (B) and IgG4 (C). The right side of each panel represents the glycoforms at the charge state +2 while the left side shows the same glycoforms at the charge state +3. The individual glycoforms are represented using the Symbol Nomenclature for Glycans. [file 13075_2022_2897_MOESM2_ESM.docx]
