# Supplementary material for: Baseline IgG-Fc N-glycosylation profile is associated with long-term outcome in a cohort of early inflammatory arthritis patients
Source: Arthritis Res Ther. 2022 Aug 25;24:206. doi: 10.1186/s13075-022-02897-5 (PMC9404591; doi:10.1186/s13075-022-02897-5)
Supplement: Supplementary file 3 — Additional file 3: Supplementary Table 1. Average relative abundances, standard deviations (SD), coefficients of variation (CV) and median CVs of IgG N-glycoforms, from the 7 serum standards, quantified with the LaCyTools software. Relative abundances were calculated following normalization of total intensity as described in the Methods section. [file 13075_2022_2897_MOESM3_ESM.docx]

| **IgG subclass** | ***N*-glycoforms** | **Average (%)** | **SD (%)** | **CV (%)** | **Median CV (%)** |
| --- | --- | --- | --- | --- | --- |
| 1 | H3N3F1 | 2.86 | 0.08 | 2.67 | 3.74 |
|  | H3N4 | 1.23 | 0.09 | 7.49 |  |
|  | H3N4F1 | 14.63 | 0.51 | 3.48 |  |
|  | H3N5F1 | 7.06 | 0.34 | 4.81 |  |
|  | H4N3F1 | 1.44 | 0.05 | 3.29 |  |
|  | H4N4 | 2.43 | 0.10 | 4.00 |  |
|  | H4N4F1 | 27.17 | 0.36 | 1.31 |  |
|  | H4N5F1 | 11.88 | 0.26 | 2.15 |  |
|  | H5N4F1 | 10.11 | 0.29 | 2.88 |  |
|  | H5N5F1 | 2.57 | 0.05 | 2.07 |  |
|  | H4N4F1S1 | 3.30 | 0.19 | 5.80 |  |
|  | H5N4S1 | 0.59 | 0.03 | 5.70 |  |
|  | H5N4F1S1 | 12.10 | 0.41 | 3.39 |  |
|  | H5N5F1S1 | 0.84 | 0.04 | 4.88 |  |
|  | H5N4F1S2 | 1.17 | 0.11 | 9.46 |  |
|  | H5N5F1S2 | 0.59 | 0.04 | 7.01 |  |
| 2/3 | H3N4F1 | 25.32 | 0.94 | 3.71 | 4.88 |
|  | H3N5F1 | 9.43 | 0.47 | 4.97 |  |
|  | H4N3F1 | 1.48 | 0.12 | 8.00 |  |
|  | H4N4F1 | 24.39 | 0.53 | 2.19 |  |
|  | H4N5F1 | 8.35 | 0.39 | 4.72 |  |
|  | H5N4F1 | 8.30 | 0.18 | 2.20 |  |
|  | H5N5F1 | 2.12 | 0.19 | 9.06 |  |
|  | H4N4F1S1 | 8.04 | 0.63 | 7.80 |  |
|  | H5N4F1S1 | 12.58 | 0.61 | 4.88 |  |
| 4 | H3N3F1 | 3.07 | 0.13 | 4.31 | 4.10 |
|  | H3N4F1 | 18.03 | 0.62 | 3.46 |  |
|  | H3N5F1 | 8.92 | 0.40 | 4.52 |  |
|  | H4N3F1 | 1.24 | 0.07 | 5.55 |  |
|  | H4N4F1 | 23.36 | 0.61 | 2.63 |  |
|  | H4N5F1 | 8.89 | 0.35 | 3.89 |  |
|  | H5N4F1 | 10.31 | 0.28 | 2.68 |  |
|  | H5N5F1 | 1.61 | 0.17 | 10.62 |  |
|  | H4N4F1S1 | 7.67 | 0.35 | 4.51 |  |
|  | H5N4F1S1 | 16.90 | 0.34 | 2.02 |  |
