# Supplementary material for: Baseline IgG-Fc N-glycosylation profile is associated with long-term outcome in a cohort of early inflammatory arthritis patients
Source: Arthritis Res Ther. 2022 Aug 25;24:206. doi: 10.1186/s13075-022-02897-5 (PMC9404591; doi:10.1186/s13075-022-02897-5)
Supplement: Supplementary file 4 — Additional file 4: Supplementary Table 2. Association of relative abundances of IgG N-glycoforms and the two diagnosis groups. Standard errors, p-values and p-values adjusted for multiple testing (p-value adjusted > 0.05) were calculated following the general linear model with age, sex and duration of symptoms included as additional covariates. [file 13075_2022_2897_MOESM4_ESM.docx]

| **IgG *N*-glycoforms** | **Effect** | **Standard errors** | **p-values** | **p-values adjusted** |
| --- | --- | --- | --- | --- |
| IgG1 H4N4F1 | 0.38623 | 0.19699 | 0.04477 | 0.44845 |
| IgG4 H5N4F1S1 | 0.30496 | 0.19402 | 0.10626 | 0.44845 |
| IgG2/3 H5N4F1 | 0.26552 | 0.17401 | 0.11673 | 0.44845 |
| IgG4 H5N4F1 | 0.27636 | 0.19934 | 0.15360 | 0.44845 |
| IgG1 H5N4F1 | 0.22031 | 0.17193 | 0.18685 | 0.44845 |
| IgG1 H5N4F1S1 | 0.15547 | 0.16870 | 0.34133 | 0.68267 |
| IgG2/3 H5N4F1S1 | 0.11041 | 0.16312 | 0.48415 | 0.82996 |
| IgG4 H3N4F1 | -0.08228 | 0.20119 | 0.67223 | 0.84571 |
| IgG4 H4N4F1 | -0.09080 | 0.22482 | 0.67606 | 0.84571 |
| IgG2/3 H3N4F1 | -0.06387 | 0.17441 | 0.70476 | 0.84571 |
| IgG1 H3N4F1 | 0.04760 | 0.18292 | 0.78772 | 0.85933 |
| IgG2/3 H4N4F1 | 0.01062 | 0.22035 | 0.96020 | 0.96020 |
