# Supplementary material for: Baseline IgG-Fc N-glycosylation profile is associated with long-term outcome in a cohort of early inflammatory arthritis patients
Source: Arthritis Res Ther. 2022 Aug 25;24:206. doi: 10.1186/s13075-022-02897-5 (PMC9404591; doi:10.1186/s13075-022-02897-5)
Supplement: Supplementary file 5 — Additional file 5: Supplementary Table 3. Association of the relative abundances of IgG N-glycoforms of the two diagnosis groups and the different clinical outcomes (“favorable outcome” and “adverse outcomes 1” and “2”). Standard errors, p-values and p-values adjusted for multiple testing were calculated based on the general linear model with age, sex and duration of symptoms included as additional covariates. Statistically significant associations are described by an adjusted p-value < 0.05. [file 13075_2022_2897_MOESM5_ESM.docx]

| **IgG**  ***N*-glycoforms** | **Clinical outcome** | **Effect** | **Standard**  **errors** | **p-values** | **p-values**  **adjusted** |
| --- | --- | --- | --- | --- | --- |
| IgG2/3 H5N4F1 | Favorable outcome | 0.62709 | 0.19178 | 0.00101 | 0.03623 |
| IgG2/3 H3N4F1 | Favorable outcome | -0.55122 | 0.18648 | 0.00276 | 0.04963 |
| IgG2/3 H5N4F1S1 | Favorable outcome | 0.46987 | 0.17652 | 0.00669 | 0.08028 |
| IgG1 H5N4F1 | Favorable outcome | 0.47524 | 0.1956 | 0.01292 | 0.09722 |
| IgG1 H4N4F1 | Adverse outcome 1 | -0.67354 | 0.28259 | 0.01523 | 0.09722 |
| IgG1 H5N4F1S1 | Favorable outcome | 0.43845 | 0.18686 | 0.01620 | 0.09722 |
| IgG1 H3N4F1 | Favorable outcome | -0.45090 | 0.20351 | 0.02289 | 0.10302 |
| IgG2/3 H5N4F1 | Adverse outcome 1 | -0.54348 | 0.24873 | 0.02571 | 0.10302 |
| IgG1 H4N4F1 | Adverse outcome 2 | -0.58699 | 0.27043 | 0.02576 | 0.10302 |
| IgG1 H5N4F1 | Adverse outcome 1 | -0.49337 | 0.24581 | 0.04004 | 0.14112 |
| IgG1 H5N4F1 | Adverse outcome 2 | -0.46203 | 0.23543 | 0.04312 | 0.14112 |
| IgG1 H5N4F1S1 | Adverse outcome 1 | -0.44221 | 0.24098 | 0.05999 | 0.16726 |
| IgG2/3 H5N4F1 | Adverse outcome 2 | -0.43597 | 0.23975 | 0.06040 | 0.16726 |
| IgG1 H3N4F1 | Adverse outcome 1 | 0.44090 | 0.26085 | 0.08275 | 0.21278 |
| IgG1 H5N4F1S1 | Adverse outcome 2 | -0.36269 | 0.22706 | 0.09808 | 0.22546 |
| IgG2/3 H4N4F1 | Adverse outcome 2 | -0.47634 | 0.30013 | 0.10020 | 0.22546 |
| IgG4 H5N4F1 | Adverse outcome 1 | -0.41390 | 0.28871 | 0.14022 | 0.29693 |
| IgG2/3 H4N4F1 | Adverse outcome 1 | -0.44301 | 0.31572 | 0.14873 | 0.29746 |
| IgG4 H4N4F1 | Favorable outcome | -0.35303 | 0.2598 | 0.15801 | 0.29938 |
| IgG2/3 H5N4F1S1 | Adverse outcome 1 | -0.31252 | 0.23462 | 0.17020 | 0.30032 |
| IgG2/3 H3N4F1 | Adverse outcome 1 | 0.32970 | 0.25044 | 0.17519 | 0.30032 |
| IgG4 H3N4F1 | Adverse outcome 1 | 0.34730 | 0.28945 | 0.21620 | 0.35237 |
| IgG4 H3N4F1 | Favorable outcome | -0.26539 | 0.22901 | 0.22767 | 0.35237 |
| IgG4 H5N4F1 | Favorable outcome | 0.27657 | 0.24317 | 0.23631 | 0.35237 |
| IgG1 H3N4F1 | Adverse outcome 2 | 0.27651 | 0.24738 | 0.24470 | 0.35237 |
| IgG4 H5N4F1 | Adverse outcome 2 | -0.29601 | 0.28791 | 0.28424 | 0.39357 |
| IgG4 H5N4F1S1 | Adverse outcome 1 | -0.26182 | 0.28390 | 0.34100 | 0.45467 |
| IgG4 H4N4F1 | Adverse outcome 2 | -0.26517 | 0.31066 | 0.37338 | 0.48006 |
| IgG2/3 H5N4F1S1 | Adverse outcome 2 | -0.16366 | 0.21916 | 0.43581 | 0.54101 |
| IgG2/3 H3N4F1 | Adverse outcome 2 | 0.15889 | 0.23420 | 0.47879 | 0.57454 |
| IgG2/3 H4N4F1 | Favorable outcome | 0.16059 | 0.25710 | 0.51396 | 0.59686 |
| IgG4 H3N4F1 | Adverse outcome 2 | 0.13026 | 0.27338 | 0.61856 | 0.69588 |
| IgG4 H5N4F1S1 | Favorable outcome | 0.09623 | 0.22541 | 0.65528 | 0.71485 |
| IgG1 H4N4F1 | Favorable outcome | 0.06102 | 0.23574 | 0.78654 | 0.83192 |
| IgG4 H4N4F1 | Adverse outcome 1 | -0.07624 | 0.32609 | 0.80881 | 0.83192 |
| IgG4 H5N4F1S1 | Adverse outcome 2 | -0.04472 | 0.26650 | 0.86071 | 0.86071 |
