# Supplementary material for: Baseline IgG-Fc N-glycosylation profile is associated with long-term outcome in a cohort of early inflammatory arthritis patients
Source: Arthritis Res Ther. 2022 Aug 25;24:206. doi: 10.1186/s13075-022-02897-5 (PMC9404591; doi:10.1186/s13075-022-02897-5)
Supplement: Supplementary file 6 — Additional file 6: Supplementary Table 4. Association of baseline clinical characteristics and long-term outcomes. Standard errors, p-values and p-values adjusted for multiple testing were determined using a general linear model (age, sex and duration of symptoms included as additional covariates) and based on HAQ, DAS28, seropositivity (presence of RF and/or anti-CCP antibodies) at baseline of the two diagnosis groups for the “favorable outcome” and “adverse outcomes 1” and “2”. Statistically significant associations are described by an adjusted p-value < 0.05. [file 13075_2022_2897_MOESM6_ESM.docx]

| **Clinical**  **characteristics** | **Clinical outcome** | **Effect** | **Standard**  **errors** | **p-values** | **p-values**  **adjusted** |
| --- | --- | --- | --- | --- | --- |
| HAQ | Adverse outcome 2 | -4.00925 | 1.52143 | 0.00040 | 0.00476 |
| HAQ | Adverse outcome 1 | -3.59950 | 1.36026 | 0.00086 | 0.00518 |
| DAS28 | Favorable outcome | 17.87952 | 2 098.43923 | 0.01230 | 0.04918 |
| Diagnosis groups | Adverse outcome 2 | -1.35962 | 0.74629 | 0.04855 | 0.12226 |
| DAS28 | Adverse outcome 2 | -17.26179 | 2 114.22939 | 0.05562 | 0.12226 |
| DAS28 | Adverse outcome 1 | -16.94314 | 1 904.02769 | 0.06900 | 0.12226 |
| Diagnosis groups | Favorable outcome | 0.98216 | 0.55667 | 0.07132 | 0.12226 |
| Seropositivity | Adverse outcome 2 | 1.26809 | 0.78228 | 0.11277 | 0.16916 |
| Diagnosis groups | Adverse outcome 1 | -0.96520 | 0.76393 | 0.18146 | 0.24194 |
| HAQ | Favorable outcome | 0.78845 | 0.71224 | 0.26420 | 0.30522 |
| Seropositivity | Favorable outcome | -0.78032 | 0.72850 | 0.27978 | 0.30522 |
| Seropositivity | Adverse outcome 1 | 0.65852 | 0.76795 | 0.40625 | 0.40625 |
