# Supplementary material for: Baseline IgG-Fc N-glycosylation profile is associated with long-term outcome in a cohort of early inflammatory arthritis patients
Source: Arthritis Res Ther. 2022 Aug 25;24:206. doi: 10.1186/s13075-022-02897-5 (PMC9404591; doi:10.1186/s13075-022-02897-5)
Supplement: Supplementary file 7 — Additional file 7: Supplementary Table 5. Association of IgG N-glycoforms and baseline clinical characteristics (HAQ, DAS28, seropositivity, acute phase reactants and autoantibody levels) of the two diagnostic groups. Standard errors, p-values and p-values adjusted for multiple testing were calculated following a general linear model (age, sex and duration of symptoms included as additional covariates) and based on the relative abundance of IgG N-glycoforms (p-value adjusted > 0.05). [file 13075_2022_2897_MOESM7_ESM.docx]

| **IgG**  ***N*-glycoforms** | **Clinical**  **characteristics** | **Effect** | **Standard**  **errors** | **p-values** | **p-values**  **adjusted** |
| --- | --- | --- | --- | --- | --- |
| IgG23_H3N4F1S0 | CRP_0 | 0.07363 | 0.02150 | 0.00060 | 0.08694 |
| IgG4_H3N4F1S0 | CRP_0 | 0.07735 | 0.02512 | 0.00191 | 0.08694 |
| IgG4_H5N4F1S0 | CRP_0 | -0.07710 | 0.02526 | 0.00209 | 0.08694 |
| IgG1_H3N4F1S0 | CRP_0 | 0.06854 | 0.02297 | 0.00259 | 0.08694 |
| IgG23_H5N4F1S1 | CRP_0 | -0.05900 | 0.02049 | 0.00358 | 0.08694 |
| IgG1_H3N4F1S0 | ESR_0 | 0.33785 | 0.11027 | 0.00201 | 0.08694 |
| IgG4_H3N4F1S0 | ESR_0 | 0.34972 | 0.12195 | 0.00373 | 0.08694 |
| IgG4_H5N4F1S1 | RF | 0.93013 | 0.32528 | 0.00384 | 0.08694 |
| IgG23_H3N4F1S0 | ESR_0 | 0.29875 | 0.10617 | 0.00440 | 0.08975 |
| IgG1_H5N4F1S1 | CRP_0 | -0.05447 | 0.02162 | 0.01040 | 0.16313 |
| IgG4_H5N4F1S1 | CRP_0 | -0.06037 | 0.02515 | 0.01448 | 0.18431 |
| IgG4_H5N4F1S0 | ESR_0 | -0.30155 | 0.12375 | 0.01313 | 0.18431 |
| IgG23_H5N4F1S1 | antiCCP | -0.49737 | 0.22363 | 0.02325 | 0.23837 |
| IgG23_H5N4F1S1 | ESR_0 | -0.22404 | 0.10087 | 0.02337 | 0.23837 |
| IgG23_H5N4F1S0 | CRP_0 | -0.04904 | 0.02257 | 0.02636 | 0.24447 |
| IgG1_H5N4F1S1 | ESR_0 | -0.22090 | 0.10509 | 0.03163 | 0.25811 |
| IgG1_H5N4F1S0 | ESR_0 | -0.21705 | 0.10742 | 0.03866 | 0.27230 |
| IgG4_H3N4F1S0 | RF | -0.67202 | 0.34081 | 0.04359 | 0.27674 |
| IgG1_H3N4F1S0 | antiCCP | 0.46438 | 0.25242 | 0.05937 | 0.29921 |
| IgG1_H5N4F1S0 | CRP_0 | -0.04085 | 0.02240 | 0.06125 | 0.29921 |
| IgG23_H5N4F1S0 | ESR_0 | -0.20628 | 0.10960 | 0.05371 | 0.29921 |
| IgG4_H4N4F1S0 | RF | -0.71290 | 0.38169 | 0.05566 | 0.29921 |
| IgG23_H3N4F1S0 | antiCCP | 0.42384 | 0.24119 | 0.07145 | 0.32391 |
| IgG1_H4N4F1S0 | RF | -0.57098 | 0.34308 | 0.08750 | 0.35108 |
| IgG23_H4N4F1S0 | RF | -0.58506 | 0.37605 | 0.10982 | 0.40006 |
| IgG4_H5N4F1S1 | ESR_0 | -0.18311 | 0.12346 | 0.12693 | 0.40666 |
| IgG1_H5N4F1S1 | antiCCP | -0.33353 | 0.23575 | 0.14545 | 0.43389 |
| IgG23_H5N4F1S0 | antiCCP | -0.34238 | 0.24536 | 0.15097 | 0.43389 |
| IgG1_H5N4F1S0 | antiCCP | -0.29583 | 0.24210 | 0.20795 | 0.46617 |
| IgG23_H5N4F1S1 | RF | 0.34775 | 0.28057 | 0.20156 | 0.46617 |
| IgG4_H5N4F1S0 | RF | 0.42379 | 0.34589 | 0.20673 | 0.46617 |
| IgG23_H3N4F1S0 | RF | -0.35227 | 0.29969 | 0.22561 | 0.48962 |
| IgG4_H4N4F1S0 | CRP_0 | 0.03293 | 0.02952 | 0.24917 | 0.49951 |
| IgG23_H5N4F1S0 | RF | 0.32348 | 0.30330 | 0.27122 | 0.51083 |
| IgG1_H5N4F1S1 | RF | 0.29791 | 0.29168 | 0.29191 | 0.53172 |
| IgG1_H3N4F1S0 | RF | -0.18218 | 0.31613 | 0.55123 | 0.78440 |
| IgG23_H4N4F1S0 | antiCCP | 0.17208 | 0.30945 | 0.56524 | 0.79235 |
| IgG4_H4N4F1S0 | antiCCP | 0.12920 | 0.31630 | 0.67258 | 0.84835 |
| IgG1_H4N4F1S0 | CRP_0 | -0.01041 | 0.02665 | 0.68569 | 0.84985 |
| IgG23 H5N4F1S1 | HAQ | 0.44261 | 0.21308 | 0.0337 | 0.85933 |
| IgG1 H4N4F1 | Diagnosis groups | 0.38623 | 0.19699 | 0.0448 | 0.85933 |
| IgG4 H5N4F1S1 | Diagnosis groups | 0.30496 | 0.19402 | 0.1063 | 0.85933 |
| IgG23 H5N4F1S1 | Seropositivity | -0.3105 | 0.20345 | 0.1167 | 0.85933 |
| IgG23 H5N4F1 | Diagnosis groups | 0.26552 | 0.17401 | 0.1167 | 0.85933 |
| IgG4 H5N4F1 | Diagnosis groups | 0.27636 | 0.19934 | 0.1536 | 0.85933 |
| IgG1 H5N4F1 | Diagnosis groups | 0.22031 | 0.17193 | 0.1869 | 0.85933 |
| IgG1 H4N4F1 | DAS28 | 0.37745 | 0.33294 | 0.2415 | 0.85933 |
| IgG4 H3N4F1 | Seropositivity | -0.282 | 0.25208 | 0.2487 | 0.85933 |
| IgG1 H4N4F1 | Seropositivity | -0.2785 | 0.25219 | 0.2547 | 0.85933 |
| IgG4 H5N4F1S1 | DAS28 | 0.33775 | 0.31844 | 0.273 | 0.85933 |
| IgG4 H4N4F1 | HAQ | -0.3122 | 0.30174 | 0.284 | 0.85933 |
| IgG23 H3N4F1 | HAQ | -0.2425 | 0.23829 | 0.2919 | 0.85933 |
| IgG23 H5N4F1 | HAQ | 0.22915 | 0.23709 | 0.3166 | 0.85933 |
| IgG1 H3N4F1 | Seropositivity | 0.21583 | 0.22955 | 0.3317 | 0.85933 |
| IgG1 H5N4F1S1 | Diagnosis groups | 0.15547 | 0.1687 | 0.3413 | 0.85933 |
| IgG23 H4N4F1 | DAS28 | -0.3198 | 0.35796 | 0.3553 | 0.85933 |
| IgG4 H5N4F1S1 | Seropositivity | 0.20998 | 0.24717 | 0.3802 | 0.85933 |
| IgG23 H5N4F1S1 | DAS28 | 0.21376 | 0.25927 | 0.3935 | 0.85933 |
| IgG1 H5N4F1S1 | Seropositivity | -0.1731 | 0.21296 | 0.4011 | 0.85933 |
| IgG4 H3N4F1 | DAS28 | -0.2517 | 0.31981 | 0.4152 | 0.85933 |
| IgG23 H3N4F1 | Seropositivity | 0.16958 | 0.21933 | 0.4244 | 0.85933 |
| IgG1 H5N4F1 | HAQ | 0.18616 | 0.24599 | 0.4323 | 0.85933 |
| IgG4 H5N4F1 | Seropositivity | 0.17535 | 0.2535 | 0.4746 | 0.85933 |
| IgG23 H5N4F1S1 | Diagnosis groups | 0.11041 | 0.16312 | 0.4842 | 0.85933 |
| IgG4 H5N4F1 | HAQ | 0.18054 | 0.26988 | 0.4873 | 0.85933 |
| IgG1 H5N4F1 | Seropositivity | -0.1402 | 0.21837 | 0.5068 | 0.85933 |
| IgG1 H5N4F1 | DAS28 | -0.1782 | 0.28567 | 0.5182 | 0.85933 |
| IgG23 H3N4F1 | DAS28 | -0.1622 | 0.28376 | 0.5537 | 0.85933 |
| IgG23 H5N4F1 | Seropositivity | -0.1124 | 0.2221 | 0.6008 | 0.85933 |
| IgG1 H5N4F1S1 | HAQ | 0.11685 | 0.23521 | 0.6057 | 0.85933 |
| IgG4 H4N4F1 | Seropositivity | -0.1409 | 0.28335 | 0.607 | 0.85933 |
| IgG23 H4N4F1 | HAQ | -0.142 | 0.29671 | 0.6191 | 0.85933 |
| IgG1 H3N4F1 | DAS28 | 0.12387 | 0.30197 | 0.6706 | 0.85933 |
| IgG4 H3N4F1 | Diagnosis groups | -0.0823 | 0.20119 | 0.6722 | 0.85933 |
| IgG4 H4N4F1 | Diagnosis groups | -0.0908 | 0.22482 | 0.6761 | 0.85933 |
| IgG1 H3N4F1 | HAQ | -0.0949 | 0.24513 | 0.6874 | 0.85933 |
| IgG23 H3N4F1 | Diagnosis groups | -0.0639 | 0.17441 | 0.7048 | 0.85933 |
| IgG1 H4N4F1 | HAQ | 0.09133 | 0.28097 | 0.7354 | 0.85933 |
| IgG4 H5N4F1 | DAS28 | 0.10789 | 0.33167 | 0.7359 | 0.85933 |
| IgG23 H5N4F1 | DAS28 | -0.0933 | 0.29004 | 0.7388 | 0.85933 |
| IgG4 H4N4F1 | DAS28 | 0.10889 | 0.36777 | 0.7588 | 0.85933 |
| IgG23 H4N4F1 | Seropositivity | -0.0737 | 0.27774 | 0.7835 | 0.85933 |
| IgG1 H3N4F1 | Diagnosis groups | 0.0476 | 0.18292 | 0.7877 | 0.85933 |
| IgG4_H5N4F1S0 | antiCCP | -0.06939 | 0.28354 | 0.80007 | 0.90843 |
| IgG1_H5N4F1S0 | RF | 0.06684 | 0.30041 | 0.81789 | 0.91675 |
| IgG4_H5N4F1S1 | antiCCP | -0.05336 | 0.27689 | 0.84192 | 0.92576 |
| IgG23_H4N4F1S0 | CRP_0 | -0.00551 | 0.02905 | 0.84408 | 0.92576 |
| IgG1_H4N4F1S0 | ESR_0 | -0.01670 | 0.12854 | 0.89293 | 0.95872 |
| IgG1 H5N4F1S1 | DAS28 | 0.01887 | 0.278 | 0.9439 | 0.9602 |
| IgG4 H5N4F1S1 | HAQ | 0.01596 | 0.265 | 0.9501 | 0.9602 |
| IgG4 H3N4F1 | HAQ | 0.01454 | 0.26362 | 0.9543 | 0.9602 |
| IgG23 H4N4F1 | Diagnosis groups | 0.01062 | 0.22035 | 0.9602 | 0.9602 |
| IgG1_H4N4F1S0 | antiCCP | 0.03281 | 0.28339 | 0.90461 | 0.96236 |
| IgG4_H3N4F1S0 | antiCCP | 0.02460 | 0.28333 | 0.92839 | 0.97333 |
| IgG4_H4N4F1S0 | ESR_0 | -0.00925 | 0.14319 | 0.94667 | 0.97333 |
| IgG23_H4N4F1S0 | ESR_0 | -0.00066 | 0.14044 | 0.99613 | 0.99913 |
